# First manic episodes triggered by the nuptial context: A case series from Tunisia

**DOI:** 10.1192/j.eurpsy.2025.1078

**Published:** 2025-08-26

**Authors:** Y. Rebei, H. Ghabi, S. Aouadi, M. Karoui, H. Nefzi, R. Kammoun, F. Ellouze

**Affiliations:** 1 Psychiatry G, Razi hospital; 2 Faculty of Medicine of Tunis, El Manar University, Tunis, Tunisia

## Abstract

**Introduction:**

Manic episodes, a defining feature of bipolar disorder, are often triggered by significant psychosocial stressors. In Tunisia, marriage carries deep religious, societal, and familial significance, creating considerable pressure that may act as a precipitating factor for the onset of bipolar disorder. This case series presents three patients who experienced their first manic episode in the immediate aftermath of their wedding.

**Objectives:**

To highlight the role of marriage as a significant trigger for the first manic episodes in patients with no previous psychiatric history, within the specific socio-cultural context of Tunisia.

**Methods:**

These case reports were compiled through clinical observations and interviews with the patients and their families. All three cases involved newlywed males who developed manic symptoms shortly after marriage, requiring hospitalization.

**Results:**

- **Case 1:** A 30-year-old male with no personal or familial psychiatric history presented with manic symptoms 5 days after his wedding. Symptoms included expansive mood, irritability, insomnia, grandiose and persecutory delusions, hyperactivity, and logorrhea. He was hospitalized and treated with lithium and risperidone. After 3 weeks, he was discharged and has remained symptom-free for 1 month.
- **Case 2:** A 33-year-old male with a paternal history of schizoaffective disorder developed mania the day after his wedding, following alcohol use and sleep deprivation. His symptoms included psychomotor agitation, destruction of objects, verbal aggression, logorrhea, tachyphemia, delusions of grandeur and persecution, insomnia, and hyperactivity. He was treated with clonazepam, lithium and haloperidol. Clonazepam was discontinued before discharge, and haloperidol after 3 months. He had been episode-free for 3 years, then he had a manic episode and was hospitalized for 2 weeks. Now he’s been symptom-free for 2 months
- **Case 3:** A 31-year-old male with no personal or familial psychiatric history presented with manic symptoms 7 days after his wedding, including insomnia, expansive mood, grandiose delusions with auditory hallucinations (he believed God told him to have a son who would be the next prophet), hyperactivity, and logorrhea. He was treated with diazepam, lithium, and risperidone. He was discharged after 4 weeks, with diazepam discontinued before discharge and risperidone after 6 months. He has remained episode-free for 1 year.

**Conclusions:**

These cases highlight the significant cultural pressures surrounding marriage in Tunisia, which can serve as a potent trigger for manic episodes in individuals without prior psychiatric history. Early identification and intervention with antipsychotics and mood stabilizers proved effective in all cases. These findings emphasize the importance of psychiatric vigilance in similar socio-cultural settings to manage the onset of bipolar disorder in response to life stressors.

**Disclosure of Interest:**

None Declared

